# Isolation and Identification of Microorganisms and Antibiotic Resistance Microorganisms from Beehives Located in Palm, Corn and Longan Plantations, Thailand

**DOI:** 10.3390/microorganisms11122855

**Published:** 2023-11-24

**Authors:** Sirikwan Dokuta, Sumed Yadoung, Peerapong Jeeno, Sayamon Hongjaisee, Phadungkiat Khamnoi, Sirinya Manochomphu, Khanchai Danmek, Jakkrawut Maitip, Bajaree Chuttong, Surat Hongsibsong

**Affiliations:** 1 School of Health Sciences Research, Research Institute for Health Sciences, Chiang Mai University, Chiang Mai 50200, Thailand; sirikwan_d@cmu.ac.th (S.D.); peerapong_jeen@cmu.ac.th (P.J.); sayamon.ho@cmu.ac.th (S.H.); 2 Environmental Sciences Program, Faculty of Sciences, Chiang Mai University, Chiang Mai 50200, Thailand; sumed_y@cmu.ac.th; 3 Microbiology Unit, Diagnostic Laboratory, Maharaj Nakorn Chiang Mai Hospital, Faculty of Medicine, Chiang Mai University, Chiang Mai 50200, Thailand; micromedcmu@hotmail.com (P.K.); sirinyam_1982@hotmail.com (S.M.); 4 School of Agriculture and Natural Resources, University of Phayao, Phayao 56000, Thailand; khanchai.da@up.ac.th; 5 Faculty of Science, Energy and Environment, Rayong Campus, King Mongkut’s University of Technology North Bangkok, Bankhai 21120, Thailand; jakkrawut.m@sciee.kmutnb.ac.th; 6 Meliponini and Apini Research Laboratory, Department of Entomology and Plant Pathology, Faculty of Agriculture, Chiang Mai University, Chiang Mai 50200, Thailand; 7 Environmental, Occupational Health Sciences and NCD Center of Excellence, Research Institute for Health Sciences, Chiang Mai University, Chiang Mai 50200, Thailand

**Keywords:** antibiotic resistance, microorganisms, beehive, plantations in Thailand

## Abstract

This study aims to determine the prevalence of microorganisms and antibiotic-resistant microorganisms in beehives located on different plantations in Thailand. Seventeen swabs immersed in transport media were utilized for samples from different zones within beehives. Traditional microbial culture-based methods, biochemical tests, MALDI-TOF MS (VITEK^®^ MS, bioMerieux, Marcy-l’Étoile, France), and antibiotic drug susceptibility (disk-diffusion) tests were used to detect microorganism and antimicrobial resistance bacteria. The results from 16 beehive swabs found Gram-positive bacteria at 59.5%, Gram-negative bacteria at 35.1%, and fungi (yeast) at 5.4%. These organisms are classified as 11, 11, and 2 types of Gram-positive bacteria, Gram-negative bacteria, and fungi (yeast), respectively. Furthermore, no organism showed resistance to vancomycin or cefoxitin for antibiotic drug susceptibility testing. In contrast, all *Acinetobacter* spp. were susceptible to ciprofloxacin, levofloxacin, ceftazidime, cefotaxime, imipenem, and meropenem, except for *Acinetobacter schindleri*, which was resistant to ceftazidime and cefotaxime. For other organisms, due to the limitations of tests to identify some environmental microbial species, the antimicrobial susceptibility test results cannot be interpreted as resistant or susceptible to the drug for these organisms. The study’s findings will support prevention, healthcare services, and public health systems.

## 1. Introduction

Over the years, the problem of antimicrobial resistance in bacteria has become more severe and tends to increase continuously, which is a serious threat to health security. At present, the problem of drug resistance is not only affecting public health but also causing economic and social losses [1,2]. Microorganisms such as *Pseudomonas aeruginosa*, *Acinetobacter baumannii*, *Klebsiella pneumoniae*, and *Escherichia coli* are among the most threatening bacteria, significantly resistant to many antimicrobial agents, and only a small range of antibiotics, especially combinations working synergistically [3]. Efforts have been made to deal with this problem nationally and internationally for a long time. Collaboration between tripartite organizations on human, animal, and environmental health (Tri-partite), including the World Health Organization (WHO), the Office International des Epizooties (OIE), and the Agriculture Organization of the United Nations (FAO), have mandated that antimicrobial resistance be addressed. It is a major goal to develop and achieve a WHO global action plan on antimicrobial resistance [4]. The use of medicated feed in animal husbandry and the use of antibiotic drugs in plant-disease control have raised concerns about the emergence of antibiotic resistance in the agricultural sector, which may aid the spread of drug-resistant strains to humans [5]. Countries worldwide, including Thailand, have made efforts to develop and control the overuse of antibiotics in agriculture through various methods to prevent and mitigate the problem of antimicrobial resistance.

For foodborne pathogen identification, more investigations are necessary to prevent significant outbreaks. A study is necessary to locate and stop the spread of the infections before a significant outbreak. However, limited research has been conducted in Thailand on the microorganisms of beehives in agricultural regions.

Honey bees play a vital role as primary pollinators of plants [6]. Consequently, beekeeping has developed within Thailand’s agricultural community. Beekeeping demands significantly less land when compared to other livestock, and is environmentally responsible. Furthermore, beekeeping yields substantial returns by selling honey and bee-derived products to beekeepers. Honey bees make their food from plant materials [7]. These insect pollinators visit flowers time and time again to gather pollen and nectar, which they then share with other bees in their hive to make beebread and honey [8]. Beehives create a significant number of microorganisms throughout the process of making these products, including bacteria that originate from plants and other sections of the honey bee’s body [9]. In beebread, the majority of the microorganisms found in pollen come from the soil and phyllosphere, making this microorganism’s divergence even more evident [10,11]. Therefore, these uncommon organisms may affect the quality of honey and may infect honey bees and beekeepers who lack hygiene practices and mostly work with bare hands in this business. Nowadays, food safety and hygiene are more of a concern, but limited in-depth research has been conducted on microorganisms and antibiotic resistance microbial determinants from beehives.

Hence, this study aimed to determine the prevalence of microorganisms and antibiotic-resistant microorganisms within beehives in different agricultural settings in Thailand to prevent or reduce the spread of these organisms from natural sources to humans. This may affect the quality of human life and economic conditions in the future. In this study, microorganisms in beehives were examined at various locations using the methods of microorganisms culture in enrichment culture media, pathogens were identified by traditional biochemical tests and MALDI-TOF MS (VITEK^®^ MS, bioMerieux, Marcy-l’Étoile, France) machines, and antimicrobial drug susceptibility tests were performed.

## 2. Materials and Methods

### 2.1. Sample Collection

For sample collection, Amies swabs were used to swab on the outer surface area of the beehive (1 swab for 1 beehive). Then, this swab was kept in Amies agar gel transport media and transported to the laboratory at room temperature.

**Figure 1:**
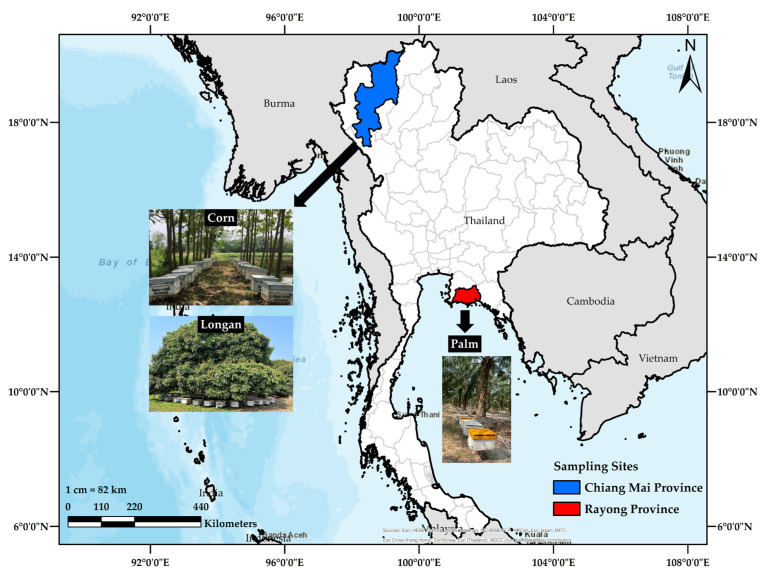
Scale map of the location of the sample plantation collection.

In March 2023, 10 Amies agar gel transport swabs were obtained from palm orchards in Rayong Province, Thailand; meanwhile, in June 2023, 1 swab was taken from a longan orchard, and 6 were collected from corn fields in Chiang Mai Province. In addition, 17 swabs were collected from various plantations in Rayong and Chiang Mai Provinces (see Figure 1).

### 2.2. Microbial Culture and Identification

Seventeen Amies agar gel transport swabs that were swabbed on a beehive were used to perform bacterial culture and identification by the standard method. Briefly, samples were cultured in sheep blood and MacConkey agar and incubated at 37 °C for 18–24 h. All suspect colonies were sub-cultured and isolated on sheep blood agar plates. Those identified were further processed using conventional methods involving the Gram staining technique and other appropriate biochemical tests, including catalase, coagulase, oxidase, indole, motility tests, sugar fermentation tests, Triple sugar iron (TSI) agar, citrate utilization, and urease production. These were performed following the laboratory standard operating procedures adopted by the Clinical and Laboratory Standard Institute (CLSI) [12,13]. In addition, a MALDI-TOF MS (VITEK^®^ MS, bioMerieux, Marcy-l’Étoile, France) machine was further used to identify the organism type. 

### 2.3. Antimicrobial Susceptibility Test

The standardized antimicrobial sensitivity test was performed on Mueller–Hinton agar (MHA) plates using the disk diffusion Kirby–Bauer technique with 0.5 McFarland turbidity standard methods, and the results were interpreted according to the Standards for Antimicrobial Susceptibility of the Clinical Laboratory Standards Institute (CLSI) protocol [12,13]. Briefly, well-isolated bacterial colonies were selected from an agar plate culture and transferred into a broth culture until a slight visible turbidity appeared (similar to the 0.5 McFarland standard). A sterile swab was dipped into the standardized suspension of bacteria, and excess fluid was removed by pressing and rotating the swab firmly against the inside of the tube above the fluid level. The swab was used to streak on the MHA plate, then antibiotic discs appropriate for organisms were placed onto the media and incubated at 37 °C for 18–24 h. After that, each plate was examined, and inhibition zones were measured to the nearest millimeter at the back of the inverted culture plate. The measurements were then compared with a standard chart adopted by the Clinical and Laboratory Standard Institute (CLSI) to determine susceptibility or resistance.

Antibiotic discs, including glycopeptide, β-lactams, fluoroquinolone, third generation cephalosporins, and the carbapenems antibiotic group, were used. They comprised vancomycin (VA30), cefoxitin (oxacillin) (CX30), ciprofloxacin (CIP5), levofloxacin (LEV5), ceftazidime (CAZ30), cefotaxime (CTX30), imipenem (IMI10), and meropenem (MEM10).

### 2.4. Data Analysis

Data are described as frequencies (counts and percentages) using Microsoft Excel.

## 3. Results

### 3.1. Microbial Culture and Identification

**Figure 2:**
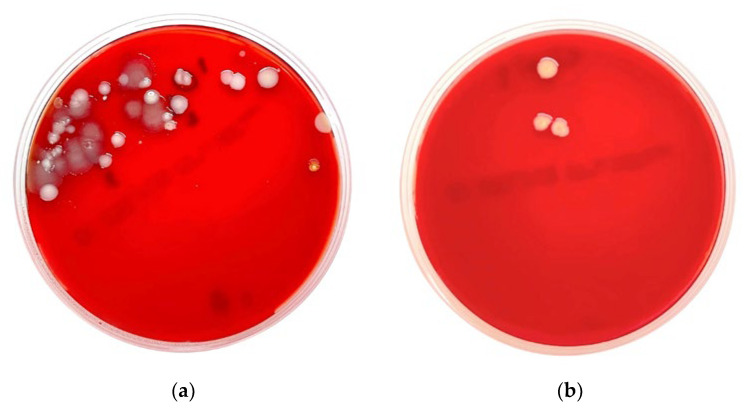
Examples of blood agar including (**a**) swab no. 16 with detection of Acinetobacter schindleri, Acinetobacter radioresistens, and Pseudomonas alcaligenesor; and (**b**) swab no. 7 with detection of Staphylococcus gallinarum and Staphylococcus sciuri.

The 17 beehive swabs from different plantations in Rayong and Chiang Mai, Thailand, were used for bacterial culture and identification by biochemical tests using MALDI-TOF MS (VITEK^®^ MS, bioMerieux, Marcy-l’Étoile, France). The results are shown in Table 1, and examples of the bacteria grown on blood agar are shown in Figure 2. One swab from the palm orchard had no growth (5.88%). The other 16 swabs showed growth (94.12%), with Gram-positive bacteria as 22 of the 37 colony sources, Gram-negative bacteria as 13 of the 37 colony sources, and fungi (yeast) as 2 of the 37 colony sources. These organisms were classified as different 11, 11, and 2 species of Gram-positive bacteria, Gram-negative bacteria, and fungi (yeast), respectively. The results of the prevalence of microbials detected on the beehives from different plantations are shown in Table 2. 

### 3.2. Antimicrobial Drug Susceptibility Test

**Figure 3:**
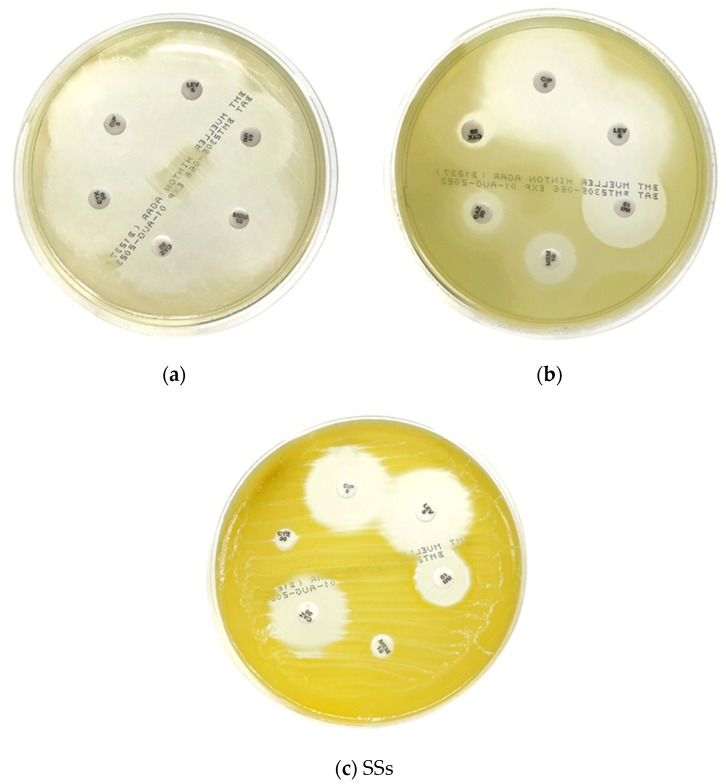
Examples of antimicrobial susceptibility tests on the MHA plate: (**a**) *Pantoea dispersa* from swab no. 11 collected from a beehive located in a longan orchard; (**b**) *Pseudomonas alcaligenes* from swab no. 16 collected from a beehive in a cornfield; (**c**) *Chryseobacterium gleum* from swab no. 17 collected from a beehive in a cornfield.

Overall, 37 organism isolates were detected on the swabs collected from the beehives. The antimicrobial susceptibility of isolates is shown in Table 3. These results were interpreted according to the Standards for Antimicrobial Susceptibility of the Clinical Laboratory Standards Institute (CLSI) protocol. Of the tested antimicrobials, no microbial organism resisted vancomycin or cefoxitin. And all Acinetobacter spp. were susceptible to ciprofloxacin (CIP5), levofloxacin (LEV5), ceftazidime (CAZ30), cefotaxime (CTX30), imipenem (IMI10), and meropenem (MEM10), except for *Acinetobacter schindleri*, which was resistant to ceftazidime (CAZ30) and cefotaxime (CTX30) (third generation cephalosporin antibiotic). The antimicrobial susceptibility test results for other organisms cannot be interpreted as resistant or susceptible to antibiotic drugs. The results provided only the inhibition zones of drug sensitivity, which were formed with each antibiotic agent (Table 3 and Figure 3).

## 4. Discussion

### 4.1. Bacterial Identification

Apiculture, or beekeeping, is widely pursued in Thailand’s agricultural sector, particularly in cultivating crops such as rambutan, longan, lychee, sunflower, palm, rubber, and other plant species. Honey bees are essential in maintaining ecological balance in natural ecosystems and providing economic advantages. Therefore, they are valuable insects for humanity. This study provided the results of the prevalence of antimicrobial resistance microorganisms found in the beehives placed in plantations of palm, longan, and corn in different provinces of Thailand.

For the results of the detection of contaminated microbials in the beehives from different plantations, we used culture-based methods and identified microbial organisms by Gram staining, traditional biochemical tests, and MALDI-TOF MS (VITEK^®^ MS, bioMerieux, Marcy-l’Étoile, France) machines. From the 17 swab specimens, we found no microbial organism growth from swab no. 5 collected from the beehives in the palm orchard [5.9% (1/17)]. This specimen’s inability to detect bacterial contamination may be because there is no microbial contamination or very low amounts of microbial contamination, along with the limitation of the sensitivity of this culture-based method [14]. In 16 other swab specimens with 37 suspected colonies, microbial contaminations were detected in samples collected from the beehives in palm orchards, longan orchards, and cornfields. We found Gram-positive bacteria at 59.5% (22/37), Gram-negative bacteria at 35.1% (13/37), and fungi (yeast) at 5.4% (2/37).

The bacteria contaminated on beehive boxes from palm orchards were 60% Gram-positive bacteria, 30% Gram-negative bacteria, and 10% yeast. *Staphylococcus* spp. and *Bacillus* spp. were the most frequently found Gram-positive bacteria. Other bacteria were *S. sciuri*, *S. gallinarum*, *B. altitudinis*/*pumilus*, *B. megaterium*, and *Exiguobacterium acetylicum*; unidentified Coryneform bacteria; and unidentified coagulase-negative *Staphylococcus* spp. Meanwhile, for Gram-negative bacteria found in the beehives placed in palm orchards, *Pantoea* spp. were detected the most frequently, followed by *A. junii* and *P. otitidis*. *C. orthopsilosis* and *C. guilliermondis*/*C. permentati* were the three types of yeast detected on the beehives located in the palm orchard.

Nevertheless, for bacterial contamination in the beehives from cornfields, 60% were Gram-positive and 40% were Gram-negative bacteria. *Bacillus* spp. were the most-detected Gram-positive bacteria, followed by unidentified Coryneform bacteria and *E. faecalis*. In contrast, *Acinetobacter* spp. were the most-detected Gram-negative bacteria, followed by *P. alcaligenes*, *C. gleum*, and unidentified Gram-negative bacteria.

For longan orchards, we obtained only 1 swab specimen collected from the beehives. We found 2 suspected organism colonies, Gram-positive and Gram-negative, with unidentified Coryneform bacteria [100% (1/1)] and *P. dispersa* [100% (1/1)], respectively.

In this study, the overall results of bacterial contamination in the beehives from different plantations indicate that we mostly detected different organisms in different areas. However, most organisms detected were Gram-positive bacteria, followed by Gram-negative bacteria and fungi (yeast).

Some detected microorganisms are an essential cause of health effects for people who work in these businesses. For Gram-positive bacteria, *Bacillus* spp. (mostly found in the beehives in palm orchards and cornfields) are thermoduric bacteria, which can produce endospores (spore-forming bacteria). Bacilli are among the major causes of food spoilage (microbial spoilage) [15]. Some strains of *Bacillus*, including *Bacillus cereus*, which was detected in the beehives placed in cornfields, are pathogens that cause food poisoning intoxication, which is caused by consuming food that contains toxins produced by these bacteria. The most well-known symptom of such a condition is gastrointestinal illness, which can be brought on by this toxin when consumed. *B. cereus*-related gastrointestinal (GI) syndromes include diarrheal illness with little upper intestine symptoms and mostly upper GI syndrome with nausea and vomiting but no diarrhea. Additionally, *B. cereus* is linked to eye and respiratory system infections, and wounds [16,17].

However, for honey bees, *Bacillus* spp. are endemic bacteria or normal flora organisms that do not cause disease. They also play a biochemical role in preserving food stored in beehives and resisting disease in bees. *Bacillus* spp. were the predominant microorganisms in the feces of worker larvae [16,18]. From the feces of 20 larvae, 44 isolates of *Bacillus* spp. were obtained. Seventeen of these were *B. megaterium*, and 19 were *B. subtilis*. *B. cereus* and *B. megaterium* were the most common *Bacillus* spp. in the intestines of queen bees [18].

The other Gram-positive bacteria detected, *Enterococcus faecalis*, found within the beehives in cornfields, is a commensal bacterium inhabiting the gastrointestinal tracts of humans [19,20]. Like other species in the genus *Enterococcus*, *E. faecalis* is found in healthy humans and can be used as a probiotic. As an opportunistic pathogen, *E. faecalis* can cause life-threatening infections, especially in the nosocomial (hospital) environment, where the naturally high levels of antibiotic resistance found in *E. faecalis* contribute to its pathogenicity [20]. *E. faecalis* can also cause endocarditis, sepsis, urinary tract infections (UTIs), meningitis, and other human infections [21,22]. Several virulent factors are thought to contribute to *E. faecalis* infections.

*Staphylococcus* spp., which was only found in the beehives collected from palm orchards, was one of the leading pathogen infections in hospitals, and many strains of this bacterium are now resistant to antibiotics. *Staphylococcus* includes at least 43 species. Many species cannot cause disease and reside normally on humans’ and other animals’ skin and mucous membranes. *Staphylococcus* species are nectar-inhabiting microbes [23]. They are also a small component of the soil microbiome [24,25].

Coryneform bacteria, or *Corynebacterium*, which could be detected in the beehives positioned in palm orchards, longan orchards, and cornfields in this study, are thermoduric bacteria, which are an important bacteriua in food and the cause of spoilage (microbial spoilage) in many types of food such as meat, poultry, etc. Furthermore, *Corynebacterium* spp. can cause a few clinically important respiratory infections, especially in immunocompromised people or those with severe respiratory disorders [26].

Additionally, we detected *Acinetobacter* spp. In the beehives in palm orchards and cornfields as Gram-negative bacteria. In nature, there are many *Acinetobacter* spp., and they frequently appear in soil and water [27]. Some *Acinetobacter* spp. can survive in a hospital setting due to their capacity to survive on both wet and dry surfaces as well as exposure to both numerous common disinfectants [27]. In nosocomial infections, Acinetobacter is commonly isolated. It is particularly common in intensive care units, where widespread sporadic cases, epidemics, and endemic occurrences occur.

The other Gram-negative bacteria, *Pantoea* spp., was detected in the beehives in palm and longan orchards. They are bacteria usually isolated from soil, water, plants, seeds, fruits (e.g., pineapples, mandarin oranges), and the gastrointestinal tracts of humans and animals in dairy products, blood, and urine. *Pantoea* spp. causes infections in humans and plants. Some are plant pathogens and opportunistic in immunocompromised humans, causing wounds, bleeding, and urinary tract inflammation [28].

*Pseudomonas* spp. and *P. alcaligenes* were detected in the beehives located in cornfields. They are Gram-negative aerobic bacteriua usually used for the bioremediation of oil pollution, pesticide substances, and certain chemical substances, as they can degrade polycyclic aromatic hydrocarbons [29]. They can be human pathogens, but such occurrences are very rare [30,31]. Whereas *P. otitidis*, which was detected in the beehives placed in palm orchards, is a new *Pseudomonas* spp. that has recently been recognized in association with otitis infections in humans, including acute otitis externa, acute otitis media, and chronic suppurative otitis media [32].

Overall, we found several organisms that may affect beekeepers and related worker health. Therefore, the beekeepers who placed their beehives in palm orchards, longan orchards, and cornfields may be concerned about the risks and dangers of these pathogen infections when handling or interacting with them. Efforts should be made to minimize the introduction of additional microorganisms into the beehives.

Nevertheless, due to the limitations of the bacterial identification test in this study, we used matrix-assisted laser desorption-ionization time of flight mass spectrometry (MALDI-TOF MS) [33] to identify microorganisms in the swabs that were collected from the beehives in different locations and plantations. We found that some organisms could not be identified. Due to the limitations of these techniques, and as technology has evolved, the expansion of databases containing spectra of known organisms has allowed for the identification of species with similar phenotypic, genotypic, and biochemical properties that were not previously possible. However, inherent similarities between organisms and a limited number of spectra in the database can lead to poor discrimination between species and misidentifications. In these cases, obtaining an incorrect species-level identification or no identification is possible. These errors occur with relatively low frequency and can typically be overcome with supplemental testing [34]. For further study, adopting high-sensitivity and specific techniques, such as molecular identification methods like 16S rRNA Gene [28,35] or next-generation sequencing technologies, is a challenging way to solve these problems and obtain more information on the study’s bacterial identification results.

### 4.2. Antimicrobial Drug Susceptibility Test

Antimicrobial drug susceptibility tests determine a microbe’s vulnerability to antimicrobial drugs by exposing a standardized concentration of organisms to specific concentrations, according to a test of a bacteria’s resistance to an antibiotic. The benefits of these laboratory tests are used to direct doctors in choosing powerful antibiotics to treat patients [36]. Susceptibility testing can be performed for bacteria, fungi, and viruses. For some organisms, the results obtained with one drug predict results with similar drugs. Thus, not all potentially useful drugs are tested.

Based on the susceptibility test of bacteria to antimicrobial agents, this study interpreted the zone of inhibition results according to the Standards for Antimicrobial Susceptibility of the Clinical Laboratory Standards Institute (CLSI) protocol [12,13]. It is a laboratory test to identify which pathogenic isolated bacteria are susceptible or resistant to any antimicrobial agent and guide physicians in selecting effective antimicrobial agents to treat patients.

Nevertheless, this study used ten antibiotic discs for susceptibility tests with the disk diffusion Kirby–Bauer technique. These antibiotic groups, including glycopeptide (vancomycin), β-lactams [cefoxitin (oxacillin)], fluoroquinolone (ciprofloxacin and levofloxacin), third generation cephalosporins (ceftazidime and cefotaxime), and carbapenems (imipenem and meropenem), are usually and commonly used as antibiotic agents with high effectiveness [37] in our setting. In addition, according to the CLSI guidelines [12,13], each organism underwent antimicrobial drug susceptibility testing against each antibiotic agent according to their Gram stain results. In this study, for Gram-positive bacteria, vancomycin and cefoxitin were tested; meanwhile, ciprofloxacin, levofloxacin, ceftazidime, cefotaxime, imipenem, and meropenem were tested for Gram-negative bacteria. Therefore, bacteria which were not tested with a specific antibiotic based on their Gram-stain result, are given ND (Not done) results in Table 3.

The antimicrobial susceptibility of isolates is shown in Table 3. This study found that 37 organism isolates were detected on swabs collected from the beehives in palm orchards, longan orchards, and cornfields. Of the antimicrobials tested for Gram-positive bacteria, all *Staphylococcus* spp., which were only found in the beehives placed in palm orchards, were resistant to cefoxitin (oxacillin), with an inhibition zone less than 25 mm (the susceptible zone diameter is ≥25 mm). According to a previous study, Chalalai et al. (2017) revealed that coagulase-negative staphylococci were the main species of *Staphylococcus* consisting of *S. gallinarum*, *S. lentus*, *S. sciuri*, *S. saprophyticus*, *S. arlettae*, *S. cohnii*, *S. simulans*, *S. carnosus*, and *S. kloosii*. They also found that the majority of isolates showed resistance to oxacillin (87%), erythromycin (52%), and clindamycin (48%) [38].

All *Acinetobacter* spp., which are Gram-negative bacteria, found in the beehives located in palm orchards and cornfields, were susceptible to ciprofloxacin (CIP5) (susceptible zone diameter is ≥25 mm), levofloxacin (LEV5) (susceptible zone diameter is ≥25 mm), ceftazidime (CAZ30) (susceptible zone diameter is ≥25 mm), cefotaxime (CTX30) (Susceptible zone diameter is ≥25 mm), and imipenem (IMI10) (susceptible zone diameter is ≥25 mm) and meropenem (MEM10) (susceptible zone diameter is ≥25 mm), except for *Acinetobacter schindleri*, which was resistant to both of the third generation cephalosporin antibiotics. Ceftazidime (CAZ30) provided an inhibition zone at 16 mm and cefotaxime (CTX30) provided an inhibition zone at 20 mm.

Moreover, for *Chryseobacterium gleum*, this detected bacteria did not form inhibition zone sizes (at 0 mm) for cefotaxime (CTX30), which is a third generation cephalosporin antibiotic drug, or meropenem (MEM10), which is a carbapenems antibiotic group. Therefore, *Chryseobacterium gleum* was resistant to these antibiotic agents.

The antimicrobial drug susceptibility tests could not interpret the other detected organisms by using the CLSI guidelines because their organism names were not specified in this guidelines. Therefore, the antimicrobial susceptibility test results cannot be interpreted as resistant or susceptible to the drug. Nevertheless, they provided a different range of inhibition zone sizes of drug sensitivity for each antibiotic agent (see Table 3). These inhibition zones represent how each antibiotic agent can inhibit bacteria growth. The size of the inhibition zone can measure the effectiveness of an antibiotic. The larger the zone of inhibition, the more bacteria were killed, indicating a higher effectiveness of the substance [39].

Regarding the bacteria in the *Bacillus* spp. group and the coryneform bacteria, which are Gram-positive, the susceptibility of antimicrobials cannot be tested by the disk diffusion method. The Minimum Inhibitory Concentration method, or MIC method, is used to quantify the minimum concentration of antimicrobial drug required to inhibit or eradicate bacteria and to test the susceptibility of the bacteria to antimicrobials for the best treatment effect [12,13,30,31].

## 5. Conclusions

Overall, microorganisms were detected in the samples swabbed from beehives placed in plantations in Rayong and Chiang Mai provinces, Thailand. These detected organisms may affect the quality of honey and their product, and may also affect beekeepers’ health if they make contact the surface area of beehives. Hence, the findings of this study can be used to disseminate knowledge to beekeepers, healthcare units, and people involved in beekeeping businesses. Beekeepers must comprehensively understand microbial infections while touching or working with these materials. The utilization of personal protection equipment and hygiene in beehives throughout the process of bee management and honey harvesting is a crucial aspect of ensuring the safety of beekeepers and their colonies.

## Figures and Tables

**Table 1:** Identification of microorganisms from beehives located in palm, corn, and longan plantations.

Swab No.	Plantation	Gram Stain Morphology	Microbial Identification	
			Biochemical Tests	MALDI-TOF MS (VITEK^®^ MS, bioMerieux, Marcy-l’Étoile, France)
1	Palm	Not performed	Yeast	*Candida orthopsilosis*
2	Palm	Gram-positive cocci	Coagulase-negative *Staphylococcus* spp.	*Staphylococcus sciuri*
		Gram-positive cocci	Coagulase-negative *Staphylococcus* spp.	Unidentified
3	Palm	Gram-positive bacilli	Coryneform bacteria	Unidentified
4	Palm	Gram-positive cocci	Coagulase-negative *Staphylococcus* spp.	*Staphylococcus sciuri*
5	Palm	No growth	No growth	Not performed
6	Palm	Gram-positive bacilli	Coryneform bacteria	Unidentified
		Gram-negative bacilli	*Pseudomonas* spp.	*Pseudomonas otitidis*
		Gram-negative bacilli	Non-fermentative Gram-negative bacilli	*Acinetobacter junii*
7	Palm	Gram-positive cocci	Coagulase-negative *Staphylococcus* spp.	*Staphylococcus gallinarum*
		Gram-positive cocci	Coagulase-negative *Staphylococcus* spp.	*Staphylococcus sciuri*
		Gram-negative bacilli	*Pantoea* spp.	*Pantoea dispersa*
8	Palm	Gram-positive bacilli with spore	*Bacillus* spp.	*Bacillus altitudinis/pumilus*
		Gram-positive bacilli	*Bacillus* spp.	*Bacillus megaterium*
		Not conducted	Yeast	*Candida guilliermondis* + *Candida permentati*
9	Palm	Gram-positive cocci	Coagulase-negative *Staphylococcus* spp.	*Staphylococcus sciuri*
		Gram-positive cocci	Coagulase-negative *Staphylococcus* spp.	*Staphylococcus gallinarum*
		Gram-negative bacilli	*Pantoea* spp.	*Pantoea ananatis*
10	Palm	Gram-positive bacilli	Coryneform bacteria	*Exiguobacterium acetylicum*
		Gram-negative bacilli	*Pantoea* spp.	Unidentified
		Gram-negative bacilli	Non-fermentative Gram negative bacilli	*Acinetobacter junii*
11	Longan	Gram-positive bacilli	Coryneform bacteria	Unidentified
		Gram-negative bacilli	*Pantoea* spp.	*Pantoea dispersa*
12	Corn	Gram-positive bacilli	Coryneform bacteria	Unidentified
13	Corn	Gram-positive bacilli	*Bacillus* spp.	*Bacillus megaterium*
		Gram-positive bacilli	Coryneform bacteria	Unidentified
14	Corn	Gram-positive bacilli	*Bacillus* spp.	Unidentified
		Gram-positive bacilli	*Bacillus* spp.	*Bacillus cereus* gr.
15	Corn	Gram-positive bacilli	*Bacillus* spp.	*Bacillus cereus* gr.
		Gram-negative cocco-bacilli	Unidentified Gram-negative bacteria	Unidentified
16	Corn	Gram-negative cocco-bacilli	Non-fermentative Gram-negative bacilli	*Acinetobacter schindleri*
		Gram-negative cocco-bacilli	Non-fermentative Gram-negative bacilli	*Acinetobacter radioresistens*
		Gram-negative bacilli	*Pseudomonas* spp.	*Pseudomonas alcaligenes*
17	Corn	Gram-positive bacilli	Coryneform bacteria	Unidentified
		Gram-negative cocco-bacilli	Non-fermentative Gram-negative bacilli	*Acinetobacter lwoffii*
		Gram-positive bacilli	*Bacillus* spp.	*Bacillus flexus*
		Gram-positive cocco-bacilli	*Enterococcus faecalis*	*Enterococcus faecalis*
		Gram-negative bacilli	Non-fermentative Gram-negative bacilli	*Chryseobacterium gleum*

**Table 2:** Prevalence of microorganism growth separated by type of plantations.

Plantation/No. of Colony Source (*n* = 37)	Type of Microorganisms		
	Gram-Positive [59.5% (22/37)]	Gram-Negative [35.1% (13/37)]	Fungus (Yeast) [5.4% (2/37)]
Palm/ (*n* = 20)	Gram-positive [60% (12/20)] *Staphylococcus sciuri* [33.3% [4/12)] *Staphylococcus gallinarum* [16.7% (2/12)] *Bacillus altitudinis/pumilus* [8.3% (1/12)] *Bacillus megaterium* [8.3% (1/12)] *Exiguobacterium acetylicum* [8.3% (1/12)] Unidentified Coagulase-negative *Staphylococcus* spp. [8.3% (1/12)] Unidentified Coryneform bacteria [16.7% (2/12)]	Gram-negative [30% (6/20)] *Acinetobacter junii* [33.3% (2/6)] *Pantoea ananatis* [16.7% (1/6)] *Pantoea dispersa* [16.7% (1/6)] *Pseudomonas otitidis* [16.7% (1/6)] Unidentified *Pantoea* spp. [16.7% (1/6)]	Fungus (Yeast) (10% (2/20)] *Candida orthopsilosis* [50% (1/2)] *Candida guilliermondis* + *Candida permentati* [50% (1/2)]
Longan/ (*n* = 2)	Gram-positive [50% (1/2)] Unidentified Coryneform bacteria [100% (1/1)]	Gram-negative [50% (1/2)] *Pantoea dispersa* [100% (1/1)]	Not Found
Corn/ (*n* = 15)	Gram-positive [60% (9/15)] *Bacillus cereus* gr. [22.2% (2/9)] *Bacillus megaterium* [11% (1/9)] *Bacillus flexus* [11% (1/9)] *Enterococcus faecalis* [11% (1/9)] Unidentified *Bacillus* spp. [11% (1/9)] Unidentified Coryneform bacteria [33.3% (3/9)]	Gram-negative [40% (6/15)] *Acinetobacter schindleri* [16.7% (1/6)] *Acinetobacter radioresistens* [16.7% (1/6)] *Pseudomonas alcaligenes* [16.7% (1/6)] *Acinetobacter lwoffii* [16.7% (1/6)] *Chryseobacterium gleum* [16.7% (1/6)] Unidentified Gram-negative bacteria [16.7% (1/6)]	Not Found

**Table 3:** Antimicrobial drug-susceptibility testing (AST) of bacterial from beehive swabs.

Swab No.	Plantation-Based Swab Source	Microorganism	Antibiotic Inhibition Zone Size (mm) ^1^							
			VA30 ^2^	CX30 ^3^	CIP5 ^4^	LEV5 ^4^	CAZ30 ^5^	CTX30 ^5^	IMI10 ^6^	MEM10 ^6^
2	Palm	*Staphylococcus sciuri*	21	23 (R)	ND	ND	ND	ND	ND	ND
		Unidentified Coagulase negative *Staphylococcus* spp.	19	22 (R)	ND	ND	ND	ND	ND	ND
4	Palm	*Staphylococcus sciuri*	20	24 (R)	ND	ND	ND	ND	ND	ND
6	Palm	*Pseudomonas otitidis*	ND	ND	38	32	18	18	19	19
		*Acinetobacter junii*	ND	ND	34 (S)	34 (S)	32 (S)	32 (S)	40 (S)	34 (S)
7	Palm	*Staphylococcus gallinarum*	19	21 (R)	ND	ND	ND	ND	ND	ND
		*Staphylococcus sciuri*	19	20 (R)	ND	ND	ND	ND	ND	ND
		*Pantoea dispersa*	ND	ND	40	36	34	30	30	34
9	Palm	*Staphylococcus sciuri*	18	22 (R)	ND	ND	ND	ND	ND	ND
		*Staphylococcus gallinarum*	19	23 (R)	ND	ND	ND	ND	ND	ND
		*Pantoea ananatis*	ND	ND	36	36	28	34	30	32
10	Palm	*Acinetobacter junii*	ND	ND	34 (S)	32 (S)	28 (S)	30 (S)	26 (S)	30 (S)
		Unidentified *Pantoea* spp.	ND	ND	22	20	21	21	34	22
11	Longan	*Pantoea dispersa*	ND	ND	38	32	26	28	26	30
15	Corn	Unidentified Gram-negative bacteria	ND	ND	44	40	34	32	32	42
16	Corn	*Acinetobacter schindleri*	ND	ND	26 (S)	26 (S)	16 (R)	20 (R)	30 (S)	28 (S)
		*Acinetobacter radioresistens*	ND	ND	26 (S)	26 (S)	23 (S)	25 (S)	28 (S)	32 (S)
		*Pseudomonas alcaligenes*	ND	ND	36	28	11	8	23	14
17	Corn	*Acinetobacter lwoffii*	ND	ND	28 (S)	28 (S)	19 (S)	24 (S)	36 (S)	30 (S)
		*Enterococcus faecalis*	20 (S)	ND	ND	ND	ND	ND	ND	ND
		*Chryseobacterium gleum*	ND	ND	24	25	19	0	14	0

Note: S: Susceptible, R: Resistant, ND: Not Done, VA30: vancomycin, CX30: cefoxitin, CIP5: ciprofloxacin, LEV5: levofloxacin, CAZ30: ceftazidime, CTX30: cefotaxime, IMI10: imipenem, MEM10: meropenem. Superscript number: ^1^ CLINICAL AND LABORATORY STANDARDS INSTITUTE^®^ M100, Performance Standards for Antimicrobial Susceptibility Testing, 32nd Edition guideline, ^2^ glycopeptide antibiotic, ^3^ β-lactams antibiotic, ^4^ fluoroquinolone antibiotic, ^5^ 3rd generation cephalosporins antibiotic, ^6^ carbapenems antibiotic.

## Data Availability

The data are contained within the article.
